# The value of a radiomics model in predicting ovarian malignancy: a retrospective multi-center comparison with O-RADS and radiologists

**DOI:** 10.1186/s13244-025-02047-w

**Published:** 2025-07-31

**Authors:** Junjie Jin, Xijia Deng, Ling Long, Meiling Liu, Meimei Cao, Hao Gong, Huan Liu, Xiaosong Lan, Lili Liu, Jiuquan Zhang

**Affiliations:** 1 Department of Radiology, Chongqing University Cancer Hospital, School of Medicine, Chongqing University, Chongqing, 400030 People’s Republic of China; 2 School of Medicine, Chongqing University, Chongqing, People’s Republic of China; 3 GE Healthcare, Medical Affairs, Shanghai, 201203 People’s Republic of China; 4 Department of Radiology, Chongqing General Hospital, Chongqing University, Shapingba, People’s Republic of China

**Keywords:** Ovarian masses, MRI, Radiomics, Ovarian-Adnexal Reporting and Data System

## Abstract

**Objectives:**

To develop an MRI–based radiomics model for ovarian masses categorization and to compare the model performance to Ovarian-Adnexal Reporting and Data System (O-RADS) and radiologists’ assessments.

**Materials and methods:**

This retrospective multicenter study included 497 patients (249 benign, 248 malignant) allocated to training, internal, and external validation sets (293/124/80 masses, respectively). Radiomics features were extracted from preoperative MRI. Features were selected using minimum redundancy, maximum relevance, and the least absolute shrinkage and selection operator algorithm. Diagnostic performance of the radiomics model, O-RADS, and independent assessments by junior and senior radiologists was evaluated via the area under the receiver operating characteristic curve (AUC) and compared using DeLong’s test.

**Results:**

In external validation, the radiomics model (AUC = 0.939) outperformed O-RADS (AUC = 0.862; *p* = 0.047) and the junior radiologist (AUC = 0.802; *p* = 0.003) and was similar to the senior radiologist (AUC = 0.886; *p* = 0.231). Subgroup analysis of O-RADS score 4 showed the model (AUC = 0.879) outperformed both radiologists (junior: *p* = 0.001; senior: *p* = 0.005). For solid, cystic–solids, and cystic masses, the AUCs of the model were 0.921, 0.975, and 0.848, respectively.

**Conclusions:**

The performance of the radiomics model to categorize ovarian masses was superior to O-RADS and junior radiologists and similar to senior radiologists. As a complementary tool to O-RADS, it allows for refined risk stratification for ovarian masses with an O-RADS score of 4 and different morphological characteristics, providing clinicians with quantitative decision support to improve preoperative diagnosis and guide treatment planning.

**Critical relevance statement:**

Radiomics model provides improved risk stratification and supports precise clinical decision-making for ovarian masses with an O-RADS score of 4 and solid, cystic-solid ovarian masses, thereby improving the management of patients with ovarian masses.

**Key Points:**

- MRI–based radiomics allows for the characterization of ovarian masses with high accuracy.
- Radiomics helps differentiate between benign and malignant ovarian masses with an O-RADS score of 4.
- For solid, cystic–solid, and cystic masses, the radiomics model exhibited higher or similar performance to that of the O-RADS and radiologists.

**Graphical Abstract:**

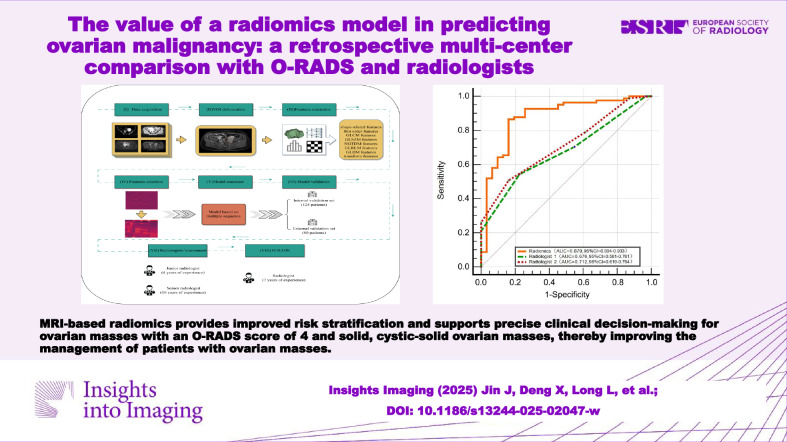

## Introduction

Ovarian cancer is the fourth most common gynecological malignancy worldwide. In 2022, there were more than 320,000 new cases of ovarian cancer and more than 200,000 deaths worldwide [1]. Accurate diagnosis of benign and malignant ovarian masses is crucial for determining appropriate treatment strategies [2]. Proper diagnosis can help avoid unnecessary surgical intervention for benign masses and optimize the surgical treatment of ovarian cancer [3, 4].

With excellent soft-tissue resolution, multi-parametric magnetic resonance imaging (MRI) is recommended for the evaluation of indeterminate ovarian masses [5–7]. The combination of conventional sequences and diffusion-weighted imaging (DWI) has been proven to have high sensitivity, specificity, and accuracy in differentiating between benign and malignant ovarian masses [8, 9]. Despite its importance in detecting ovarian masses, there are quite a few similarities in the MRI appearance of benign and malignant masses because of the complex morphological characteristics of ovarian masses [10, 11]. For instance, malignant ovarian masses can appear to have a cystic main component and resemble benign tumors, whereas benign masses can be solid or cystic–solids and mimic malignant tumors [12]. Therefore, accurately differentiating benign from malignant ovarian masses with varying morphological characteristics is critical.

Radiologists’ assessment of MR images is a routine process to classify ovarian masses. Nevertheless, because of the subjective nature of the visual analysis, qualitative assessments may vary between radiologists [13]. Hence, to improve the accuracy of the evaluation of benign and malignant adnexal lesions, the American College of Radiology introduced the Ovarian-Adnexal Reporting and Data System (O-RADS) [14], which achieves an AUC of 0.961 [15], as a standardized approach for risk stratification and management. However, managing ovarian masses with an O-RADS MRI score of 4 remains challenging because of the limited information and positive predictive value of approximately 50% [14].

Radiomics has shown promising results in clinical practice, including aiding diagnosis, treatment response evaluation, and prognosis prediction across various diseases [16–18], and has been proven to be an effective tool for classifying ovarian masses [19–28], with performance surpassing that of subjective radiologists’ assessments [21]. However, most of the studies lack external validation [19, 20] or involve small sample sizes [19], limiting their generalizability to diverse populations. Moreover, to the best of our knowledge, no prior studies have simultaneously evaluated the diagnostic performance of radiomics, O-RADS, and radiologists’ assessments in a comprehensive manner. In addition, there is a lack of research on the utility of MRI radiomics in evaluating ovarian masses with an O-RADS score of 4 and in subgroups of ovarian masses with different morphological characteristics. These gaps highlight the need for a multi-center, multi-method comparative study to define the role of radiomics in complementing O-RADS and reducing diagnostic uncertainty.

Therefore, our study aimed to develop radiomics models for categorizing ovarian masses and to compare their diagnostic performance with that of the O-RADS and radiologists’ assessment. Furthermore, we sought to explore the potential of radiomics to refine risk stratification for ovarian masses with an O-RADS score of 4 and different morphological characteristics. Our findings could significantly impact clinical practice by providing a more objective and accurate diagnostic tool, reducing unnecessary interventions, and optimizing treatment strategies for patients with ovarian masses.

## Methods

### Patients

**Fig. 1:**
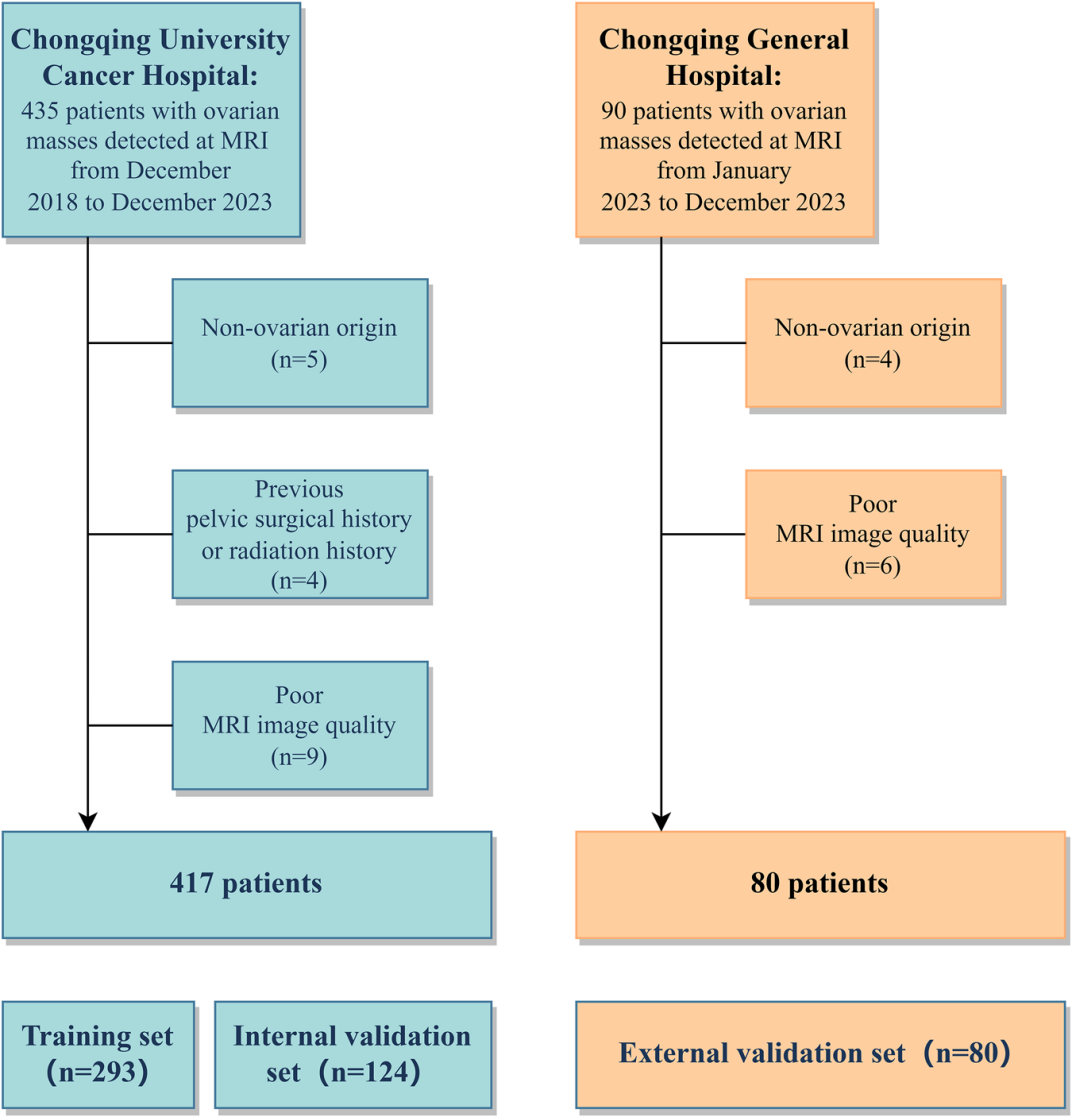
Flowchart of the study. In total, 497 of 525 patients from two clinical centers were enrolled in this study

This retrospective study was approved by our local institutional review board (CZLS2024169-A), which waived the requirement for written informed consent. Patients with clinically suspected gynecological diseases were retrospectively identified from two hospitals (Chongqing University Cancer Hospital and Chongqing General Hospital) between December 2018 and December 2023. The inclusion criteria were as follows: (1) patients who underwent a surgical procedure with histopathological results, with a time interval of no more than 30 days between MRI examination and surgery; and (2) patients who had no previous history of ovarian cancer. The exclusion criteria were as follows: (1) masses of non-ovarian origin, (2) previous pelvic surgical history or radiation history, and (3) poor MR image quality. The patient flowchart is presented in Fig. 1.

### MR image acquisition and analysis

**Fig. 2:**
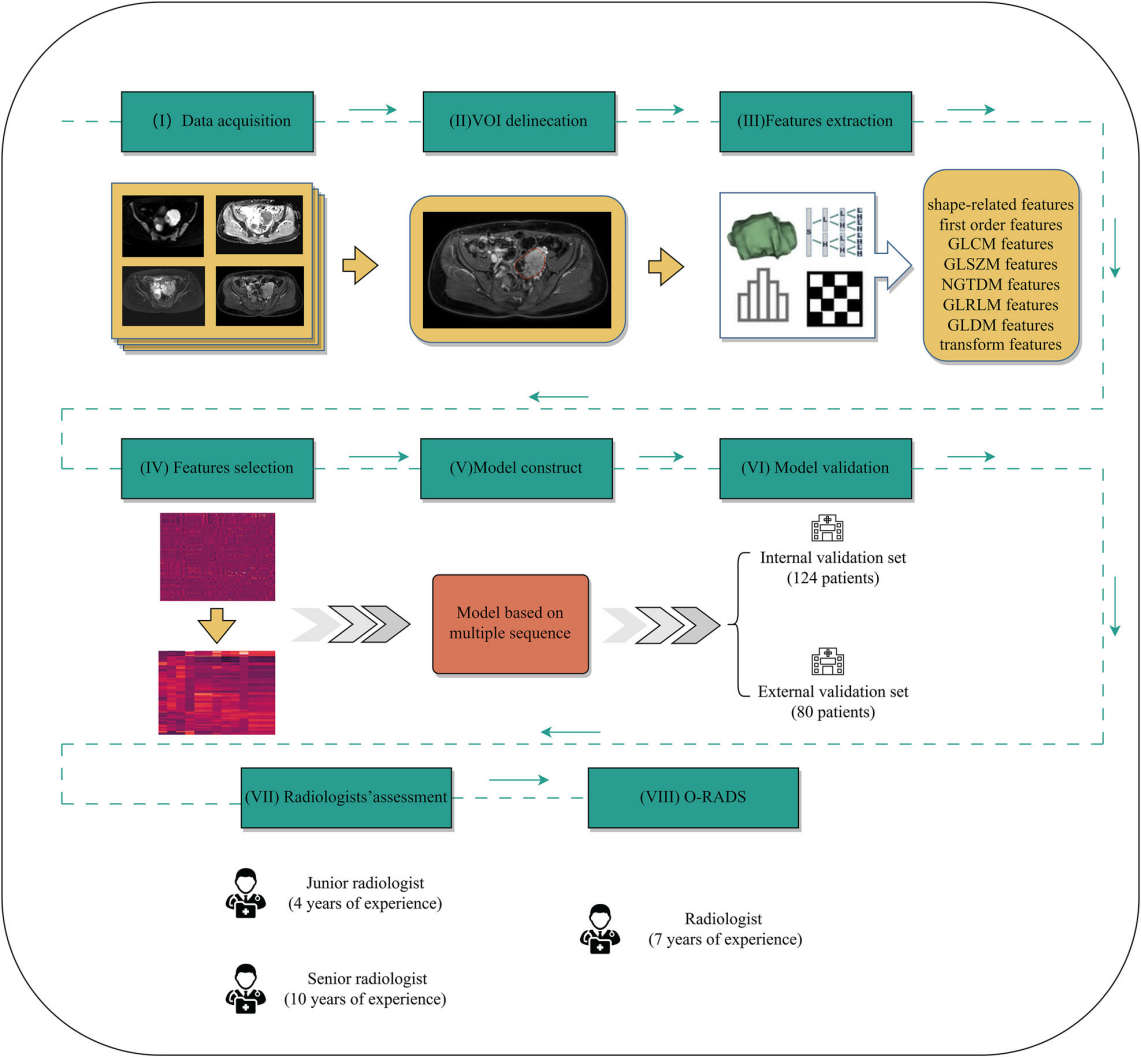
Overview of workflow, including the following steps: 1. Data Acquisition: Multi-sequence MRI from two centers. 2. Volume of Interest (VOI) Delineation: Semi-automatic VOI delineation on MRI. 3. Feature Extraction: Shape, first-order statistics, and texture features derived from both original and transformed images. 4. Feature Selection: Minimum redundancy maximum relevance (mRMR) and least absolute shrinkage and selection operator (LASSO) algorithms for feature reduction. 5. Model Construction: Logistic regression-based radiomics signature. 6. Model Validation: Internal validation (*n* = 124) and external validation (*n* = 80). 7. Radiologists’ Assessment: Independent evaluations by two radiologists (4 and 10 years’ experience). 8. O-RADS Classification: O-RADS classification per American College of Radiology (ACR) guidelines (7-year-experienced radiologist)

An overview of our workflow is illustrated in Fig. 2. All MRI examinations were performed using 1.5- or 3.0-T scanners from two manufacturers (GE Healthcare and Philips Healthcare). Data were acquired from two centers: Chongqing University Cancer Hospital utilized a 3.0-T GE SIGNA Premier scanner (*n* = 247) and a 1.5-T Philips Ingenia XD scanner (*n* = 170), while Chongqing General Hospital employed a 3.0-T Philips Ingenia DNA scanner (*n* = 80). Of the total 497 examinations, 327 (65.8%) were performed at 3.0 T, including 247 (75.5%) from GE Healthcare and 80 (24.5%) from Philips Healthcare. The remaining 170 scans (34.2%) were acquired at 1.5 T exclusively on Philips Healthcare systems. The protocol used to obtain the image at each center included the following: axial T2-weighted imaging with fat suppression (T2WI-FS), axial T2-weighted imaging without fat suppression (T2WI), sagittal T2WI without fat suppression, coronal T2WI without fat suppression, axial in- and out-of-phase T1-weighted imaging (T1WI), apparent diffusion coefficient (ADC) map, DWI (*b*-value = 0 and 1000 s/mm^2^), and gadodiamide-enhanced axial contrast-enhanced T1WI (CE-T1WI) were acquired with one precontrast and three postcontrast series in the axial plane (30-, 60-, and 150-s acquisitions). The imaging protocols for MRI are shown in Tables S1–S3.

For patients with multiple adnexal masses, the masses with the most complex morphological structure or the largest size on MRI were selected for evaluation. An experienced radiologist (L.L., with 7 years of experience in gynecological tumor MRI diagnosis), blinded to the patients’ clinical and pathological data, independently assessed the images and assigned a score to each adnexal mass based on the O-RADS MRI risk stratification system [15]. If there was solid tissue in the lesion, then the most avidly enhancing component of solid tissue observed in the first-phase enhanced images was compared with the outer myometrium of the uterus to determine whether the O-RADS score of the lesion should be 4 or 5. Before the study commenced, the radiologist underwent training in the O-RADS MRI risk stratification system. Masses with an O-RADS score ≥4 were classified as malignant.

The other two radiologists, Radiologist 1 (J.J., with 4 years of experience in pelvic MR imaging; the junior radiologist) and Radiologist 2 (X.D., with 10 years of experience in gynecological tumor MRI diagnosis; the senior radiologist), reviewed the MR images of each patient independently. They evaluated each image by assigning confidence levels to the diagnosis of malignant masses, including borderline tumors: 0, definitely benign; 0.2, probably benign; 0.4, possibly benign; 0.6, possibly malignant; 0.8, probably malignant; and 1.0, definitely malignant. Scores of 0–0.4 were classified as non-malignant, whereas scores of 0.6–1.0 were classified as malignant.

### Image segmentation and feature extraction

All Digital Imaging and Communications in Medicine images, including axial T2WI-FS, axial CE-T1WI at the third phase, ADC, and axial DWI (*b* = 1000 s/mm^2^), were exported to a commercial platform (Deepwise, version 2.5.1; https://keyan.deepwise.com). Volumes of interest (VOIs) were independently and semi-automatically generated on each sequence (axial T2WI-FS, axial CE-T1WI, ADC, and axial DWI) using Deepwise. A radiologist (J.J.) manually delineated three key axial slices of the tumor: the slice with the largest cross-sectional area and the superior and inferior boundary slices. The software then automatically propagated the contours to all remaining slices, which were reviewed and adjusted by the radiologist to ensure accuracy.

Image normalization was applied before extracting the radiomics features. Cubic spline interpolation was used to resample the voxel size, and the anisotropic voxels were resampled to form voxels of 1.0 × 1.0 × 3.0 mm (CE-T1WI) and 1.0 × 1.0 × 5.0 mm (ADC, DWI, and T2WI-FS). For each patient, radiomics features were extracted from four VOIs of MR images (DWI, ADC, T2WI-FS, and CE-T1WI), and a total of 1,688 radiomics features were extracted from each VOI using PyRadiomics in Python, including shape-related features, first-order features, texture features (gray-level co-occurrence matrix features, gray-level size zone features, gray-level run-length matrix features, neighboring gray-tone difference matrix features, and gray-level dependence matrix), and Laplace transform and wavelet transform features using corresponding VOIs.

To assess the reproducibility of the VOIs, 30 patients with a total of 120 VOIs were randomly selected for mass segmentation 1 month later by the same radiologist who was blinded to the clinical and pathological data of each patient. Additionally, a second blinded radiologist independently segmented a subset of 30 cases (120 VOIs) to evaluate interobserver variability. The intraclass correlation coefficient (ICC) was calculated to determine the stability of the features. Intra-reader ICCs were derived from repeated measurements by the primary radiologist, while inter-reader ICCs compared segmentations between the two observers. Features with intra-reader ICCs lower than 0.80 were excluded, and the remaining features were used for subsequent evaluation.

### Feature selection and model construction

To ensure the independence of the validation set and prevent data leakage during model development, the entire cohort from Chongqing University Cancer Hospital (*n* = 417) was randomly partitioned into a training set (70%, *n* = 293) and an internal validation set (30%, *n* = 124) using stratified random sampling based on mass malignancy status (benign vs. malignant). All subsequent preprocessing, feature selection, and model-building steps were performed exclusively on the training set.

Before the feature-selection process and exclusively on the training set, the abnormal and missing values were replaced by the median, and features were normalized by z-score normalization. Within the training set, the minimum redundancy and maximum relevance, and the least absolute shrinkage and selection operator regression, incorporating 10-fold cross-validation, were then applied to select significant features. Next, stepwise logistic regression analysis with the Akaike information criterion was used to select the most optimal malignant-related ovarian cancer features. Finally, a radiomics score was calculated using the training set with a formula incorporating the selected features that were weighted by their respective coefficients derived from the training set. The potential association with ovarian cancer was initially assessed in the training set, and the final model was then strictly applied without any retraining or parameter adjustment to the internal validation and the independent external test sets for subsequent validation.

### Reference standard

The final diagnosis was determined by histopathological examination following surgical resection. According to the World Health Organization’s International Classification of Diseases for Oncology, masses were classified as benign, borderline, or malignant. For analyses, borderline ovarian tumors were classified as malignant [15, 29, 30].

### Statistical analysis

All statistical analyses were conducted using MedCalc® Statistical Software version 20.022 (MedCalc Software, Ltd.; https://www.medcalc.org; 2021). The normality of the distribution of continuous variables was tested using the Kolmogorov–Smirnov test. Continuous variables with normal distribution were presented as mean ± standard deviation and ranges. Continuous variables with non-normal distribution were presented as medians and first (Q1) and third (Q3) quartiles. Receiver operating characteristic (ROC) curves were generated to evaluate predictive performance, with optimal thresholds determined by maximizing Youden’s index on the training set and fixed for validation. The area under the ROC curve (AUC) and corresponding 95% confidence interval (CI) were obtained from ROC curves, along with the sensitivity and specificity. Confidence intervals were computed using DeLong’s method for AUC and the Wilson score method with continuity correction for binomial proportions (sensitivity/specificity). The diagnostic performance of the radiomics model, O-RADS, and radiologists’ assessments was evaluated using the AUC. AUCs were compared using DeLong’s test.

Subgroup analyses were performed as post-hoc analyses to assess the robustness of the radiomics model in specific clinical contexts and technical settings. Clinical subgroups included masses with O-RADS score 4 and masses categorized by morphological subtype (solid, cystic-solid, and cystic). Technical heterogeneity subgroups were defined by magnetic field strength (1.5 T vs. 3.0 T) and scanner manufacturer (GE vs. Philips). In all subgroup analyses, AUCs were compared using DeLong’s test.

*p* < 0.05 was considered statistically significant.

## Results

### Patient characteristics

A total of 497 patients (mean age, 48.1 ± 14.7 years; age range, 14–81 years) were enrolled in this study. Among them, 249 patients (50.1%) had benign masses, and 248 patients (49.9%) had malignant masses. The baseline characteristics of the study population are summarized in Table 1. The training set had 293 patients (141 benign masses and 152 malignant masses), the internal validation set had 124 patients (60 benign masses and 64 malignant masses), and the external validation set had 80 patients (48 benign masses and 32 malignant masses). The detailed histopathology results for the ovarian masses are summarized in Table S4.

**Table 1:** Demographic and clinicopathologic characteristics of patients with ovarian masses

	Training set (*n* = 293)	Internal validation set (*n* = 124)	External validation set (*n* = 80)	Total (*n* = 497)
Benign	141 (48.1)	60 (48.4)	48 (60.0)	249 (50.1)
Malignant	152 (51.9)	64 (51.6)	32 (40.0)	248 (49.9)
Age (years)^a^	47.4 ± 14.7 (14–81)	49.9 ± 14.0 (17–79)	48.0 ± 15.6 (18–81)	48.1 ± 14.7 (14–81)
CA125 (U/mL)	41.9 (12.4–353.4)	53.2 (14.7–378.7)	30.4 (14.2–154.4)	40.3 (13.1–329.8)
Hormonal status				
Premenopausal	141 (48.1)	68 (54.8)	45 (56.3)	254 (51.1)
Postmenopausal	152 (51.9)	56 (45.2)	35 (43.7)	243 (48.9)
Maximum diameter (cm)	7.8 (4.9–11.0)	7.9 (4.9–12.9)	7.2 (4.4–9.9)	7.7 (4.9–11.1)
Location				
Unilateral	222 (75.8)	98 (79)	60 (75.0)	380 (76.5)
Bilateral	71 (24.2)	26 (21)	20 (25.0)	117 (23.5)
Pathological type				
Epithelial	188 (64.2)	85 (68.5)	41 (51.3)	314 (63.2)
Sex cord stromal	16 (5.5)	6 (4.8)	5 (6.2)	27 (5.4)
Germ cell	69 (23.5)	23 (18.6)	14 (17.5)	106 (21.3)
Others	20 (6.8)	10 (8.1)	20 (25.0)	50 (10.1)
Lesion morphology				
Solid	60 (20.5)	28 (22.6)	13 (16.3)	101 (20.3)
Cystic-solid	163 (55.6)	61 (49.2)	34 (42.5)	258 (51.9)
Cystic	70 (23.9)	35 (28.2)	33 (41.2)	138 (27.8)

Categorical variables are shown as numbers, and data in parentheses are percentages. Data are medians; data in parentheses are IQRs

*CA-125* cancer antigen 125

^a^ Data are means ± standard deviation (SD)

### Model development and performance

**Fig. 3:**
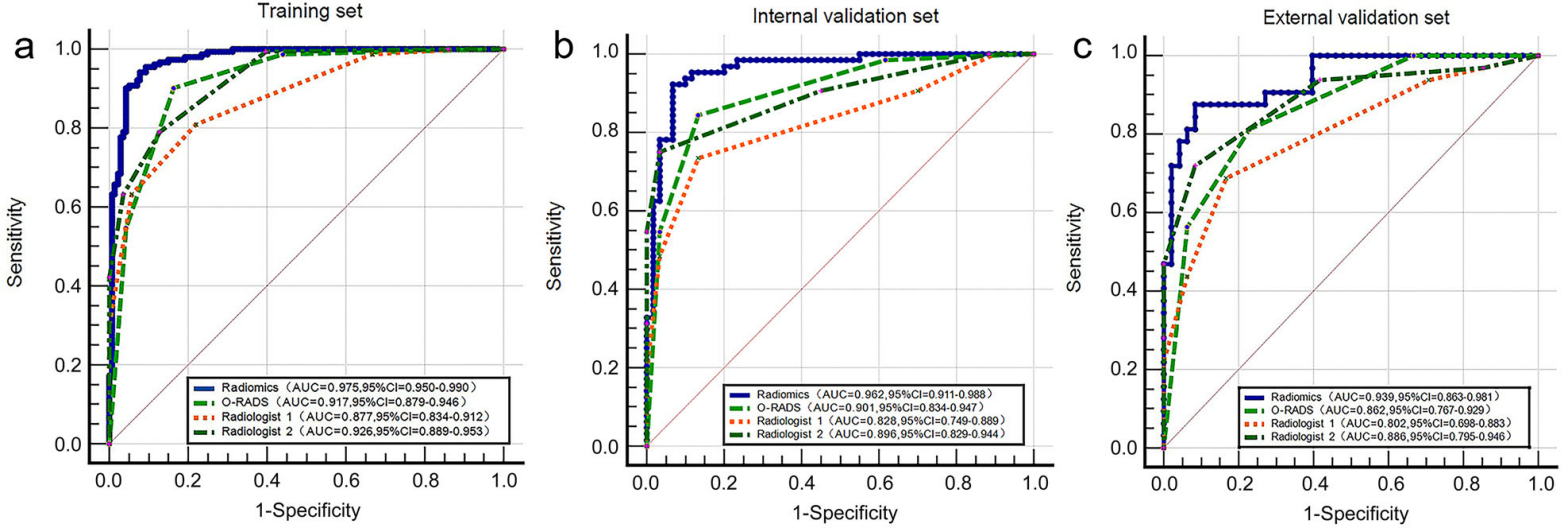
Receiver operating characteristic curves of the radiomics model, Ovarian-Adnexal Reporting and Data System, and radiologists’ assessments across datasets. **a** Training set: Performance on the training cohort (*n* = 293). **b** Internal validation set: Performance on the internal validation cohort (*n* = 124). **c** External validation set: Performance on the independent external cohort (*n* = 80). The diagonal dashed line indicates the reference for random chance (AUC = 0.5)

The mean intra-reader ICC value for the radiomics features was 0.874, indicating high repeatability, while the inter-reader ICC showed moderate agreement (mean = 0.78, range 0.65–0.92). Following feature selection, 16 robust features were retained, the ultimately selected radiomics features are detailed in the Supplementary Note. The radiomics model was developed based on the multi-sequence combination (DWI + ADC + T2WI-FS + CE-T1WI). Table 2 and Fig. 3 present the results of the training set, internal validation set, and external validation set for the radiomics model. The results indicated that the combined model demonstrated robust performance in differentiating between benign and malignant ovarian masses. In the training set, the model achieved an AUC of 0.975 (95% CI: 0.950, 0.990), with a sensitivity of 0.941 and specificity of 0.922, indicating excellent diagnostic accuracy and a balanced ability to correctly identify both malignant and benign lesions. In the internal validation set, the model maintained high performance, with an AUC of 0.962 (95% CI: 0.911, 0.988), sensitivity of 0.922, and specificity of 0.933, demonstrating its generalizability within the same population. In the external validation set, the model showed slightly reduced but still strong performance, with an AUC of 0.939 (95% CI: 0.863, 0.981), sensitivity of 0.875, and specificity of 0.917. The slight decrease in sensitivity may reflect the heterogeneity of the external cohort, while the high specificity suggests the model’s ability to minimize false-positive diagnoses.

**Table 2:** Diagnostic performances and pairwise comparisons (DeLong’s test) of Radiomics, O-RADS, and Radiologists’ Assessment for classifying benign and malignant ovarian masses

Model	Training set (*n* = 293)					Internal validation set (*n* = 124)					External validation set (*n* = 80)				
	*p-*value	AUC	Sensitivity	Specificity	Threshold	*p-*value	AUC	Sensitivity	Specificity	Threshold	*p-*value	AUC	Sensitivity	Specificity	Threshold
**Radiomics**		0.975 (0.950–0.990)	0.941 (0.891–0.973)	0.922 (0.865–0.960)	> 0.182		0.962 (0.911–0.988)	0.922 (0.827–0.974)	0.933 (0.838–0.982)	> 0.182		0.939 (0.863–0.981)	0.875 (0.710–0.965)	0.917 (0.800–0.977)	> 0.182
vs. O-RADS	0.001					0.046					0.047				
vs. Radiologist 1	< 0.001					0.001					0.003				
vs. Radiologist 2	0.001					0.034					0.231				
**O-RADS**		0.917 (0.879–0.946)	0.901 (0.842–0.944)	0.837 (0.765–0.894)	> 3		0.901 (0.834–0.947)	0.844 (0.731–0.922)	0.867 (0.754–0.941)	> 3		0.862 (0.767–0.929)	0.813 (0.636–0.928)	0.771 (0.627–0.880)	> 3
vs. Radiologist 1	0.048					0.037					0.160				
vs. Radiologist 2	0.545					0.865					0.538				
**Radiologist 1**		0.877 (0.834–0.912)	0.809 (0.738–0.868)	0.780 (0.703–0.845)	> 0.4		0.828 (0.749–0.889)	0.734 (0.609–30.837)	0.867 (0.754–0.941)	> 0.4		0.802 (0.698–0.883)	0.688 (0.500–0.839)	0.833 (0.698–0.925)	> 0.4
vs. Radiologist 2	0.004					0.009					0.015				
**Radiologist 2**		0.926 (0.889–0.953)	0.790 (0.716–0.851)	0.872 (0.806–0.923)	> 0.4		0.896 (0.829–0.944)	0.750 (0.626–0.850)	0.967 (0.885–0.996)	> 0.4		0.886 (0.795–0.946)	0.719 (0.533–0.863)	0.917 (0.800–0.977)	> 0.4

Data in parentheses are 95% confidence intervals

*AUC* area under the receiver operating characteristic curve, *O-RADS* Ovarian-Adnexal Reporting and Data System

### O-RADS

As demonstrated in Table 2 and Fig. 3, the O-RADS system to characterize ovarian masses achieved high accuracy with AUCs of 0.917 (95% CI: 0.879, 0.946) for the training set, 0.901 (95% CI: 0.834, 0.947) for the internal validation set, and 0.862 (95% CI: 0.767, 0.929) for the external validation set.

### Radiologists’ assessment

Compared with Radiologist 1, Radiologist 2 achieved higher accuracy in the classification of ovarian masses; the detailed results are provided in Table 2. Radiologist 1 achieved AUCs of 0.877 (95% CI: 0.834, 0.912), 0.828 (95% CI: 0.749, 0.889), and 0.802 (95% CI: 0.698, 0.883) for the training set, internal validation set, and external validation set, respectively. Radiologist 2 achieved AUCs of 0.926 (95% CI: 0.889, 0.953), 0.896 (95% CI: 0.829, 0.944), and 0.886 (95% CI: 0.795, 0.946) for the training set, internal validation set, and external validation set, respectively. The ROC curves are presented in Fig. 3.

### Comparison of radiomics model, O-RADS, and radiologists’ assessment

As depicted in Fig. 3 and Table 2, the radiomics model demonstrated superior performance in differentiating between benign and malignant ovarian masses. In the training set, the radiomics model achieved the highest AUC, which was significantly higher than that of the O-RADS (*p* = 0.001), Radiologist 1 (*p* < 0.001), and Radiologist 2 (*p* = 0.001). In the internal validation set, the radiomics model also achieved the highest AUC, outperforming the O-RADS (*p* = 0.046), Radiologist 1 (*p* = 0.001), and Radiologist 2 (*p* = 0.034). In the external validation set, the AUC of the radiomics model was higher than that of the O-RADS (*p* = 0.047) and Radiologist 1 (*p* = 0.003), with no significant difference compared with Radiologist 2 (*p* = 0.231).

### Subgroup analysis

**Fig. 4:**
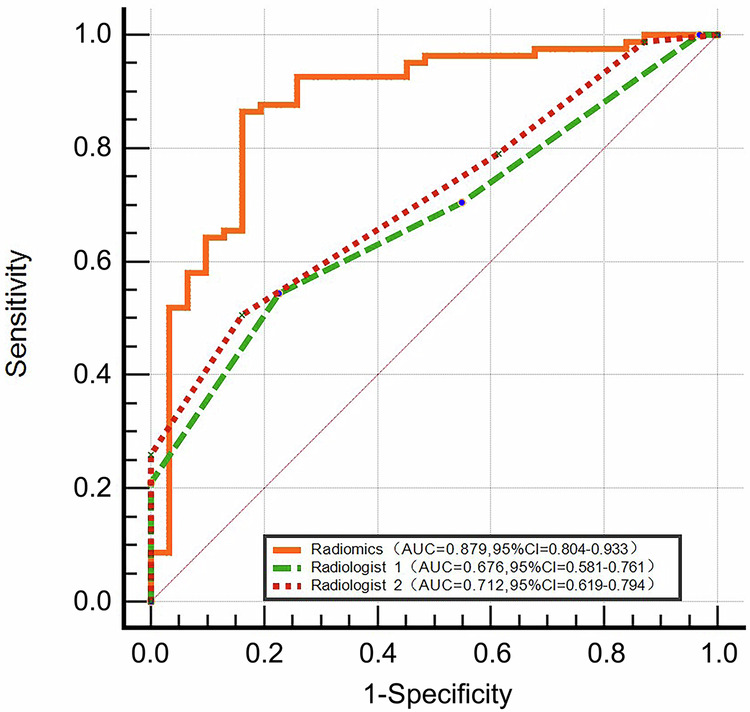
Receiver operating characteristic curves of the radiomics model and radiologists’ assessment in the subgroup of O-RADS score 4 (*n* = 112). ROC, receiver operating characteristic; O-RADS, Ovarian-Adnexal Reporting and Data System

Subgroup analyses were conducted on the combined cohort (training + internal validation +  external validation sets). Among ovarian masses assigned an O-RADS score of 4 (*n* = 112, 31 benign masses and 81 malignant masses), the radiomics model demonstrated excellent predictive performance (AUC = 0.879; 95% CI: 0.804, 0.933) in differentiating between benign and malignant ovarian masses, outperforming Radiologist 1 (AUC = 0.676; 95% CI: 0.581, 0.761; *p* = 0.001) and Radiologist 2 (AUC = 0.712; 95% CI: 0.619, 0.794; *p* = 0.005). The detailed results are presented in Fig. 4 and Table 3.

**Table 3:** Diagnostic performances and pairwise comparisons (DeLong’s test) of Radiomics, O-RADS, and Radiologists’ Assessment for classifying benign and malignant ovarian masses in the subgroup of O-RADS score 4 (*n* = 112)

Model	*p*-value	AUC	Sensitivity	Specificity
**Radiomics**		0.879 (0.804–0.933)	0.864 (0.770–0.930)	0.839 (0.663–0.945)
vs. Radiologist 1	0.001			
vs. Radiologist 2	0.005			
**Radiologist 1**		0.676 (0.581–0.761)	0.543 (0.429–0.654)	0.774 (0.589–0.904)
vs. Radiologist 2	0.046			
**Radiologist 2**		0. 712 (0.619–0.794)	0.506 (0.393–0.619)	0.839 (0.663–0.945)

Data in parentheses are 95% confidence intervals

*AUC* area under the receiver operating characteristic curve

**Fig. 5:**
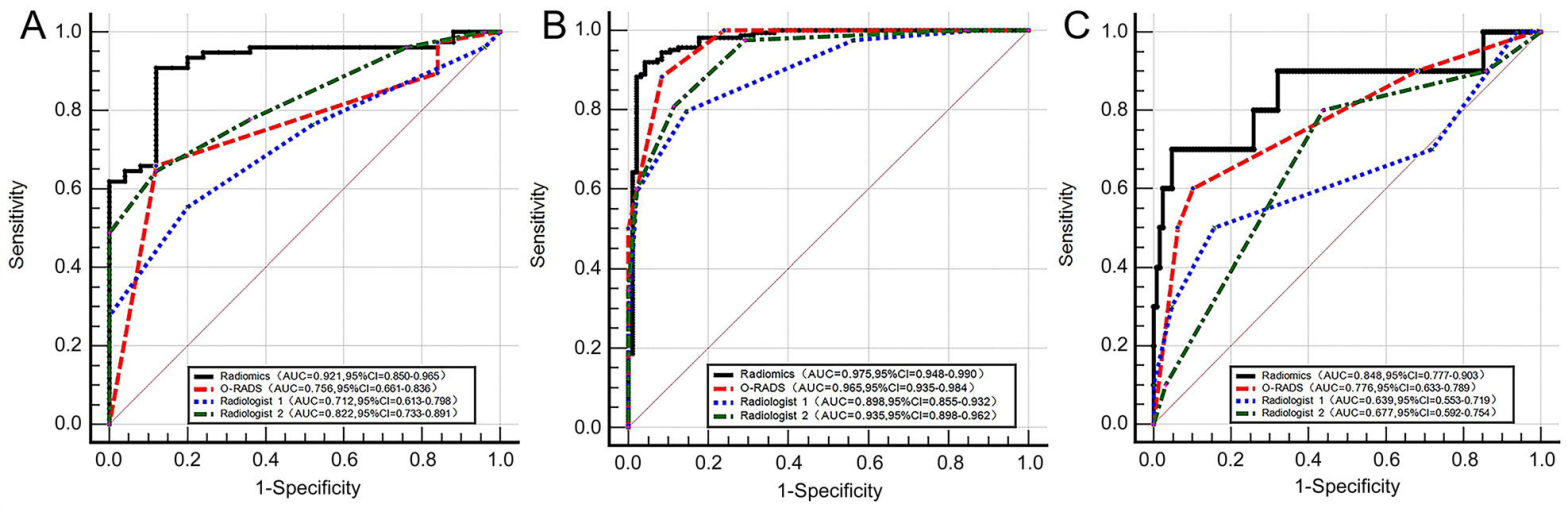
Receiver operating characteristic curves of the radiomics model, Ovarian-Adnexal Reporting and Data System (O-RADS), and radiologists’ assessments in morphological subgroups. **A** Solid masses (*n* = 101): Subgroup with predominantly solid components. **B** Cystic–solid masses (*n* = 258): Subgroup with mixed cystic and solid components. **C** Cystic masses (*n* = 138): Subgroup with predominantly cystic components. The diagonal dashed line indicates the reference for random chance (AUC = 0.5)

Further subgroup analyses were conducted based on the morphological characteristics of ovarian masses, as presented in Fig. 5 and Table 4. In the subgroup of solid masses (*n* = 101, 25 benign masses and 76 malignant masses), the radiomics model achieved the highest AUC (0.921; 95% CI: 0.850, 0.965), significantly higher than that of the O-RADS (AUC = 0.756; 95% CI: 0.661, 0.836; *p* = 0.002), Radiologist 1 (AUC = 0.712; 95% CI: 0.613, 0.798; *p* = 0.001), and Radiologist 2 (AUC = 0.822; 95% CI: 0.733, 0.891; *p* = 0.028). In the subgroup of cystic–solid masses (*n* = 258, 96 benign masses and 162 malignant masses), the radiomics model achieved an AUC of 0.975 (95% CI: 0.948, 0.990), higher than that of Radiologist 1 (AUC = 0.898; 95% CI: 0.855, 0.932; *p* < 0.001) and Radiologist 2 (AUC = 0.935; 95% CI: 0.898, 0.962; *p* = 0.008), and there was no difference between radiomics and the O-RADS (AUC = 0.965; 95% CI: 0.935, 0.984; *p* = 0.332). In the subgroup of cystic masses (*n* = 138, 128 benign masses and 10 malignant masses), there was no difference between the radiomics model (AUC = 0.848; 95% CI: 0.777, 0.903) and the O-RADS (AUC = 0.776; 95% CI: 0.633, 0.789; *p* = 0.542), Radiologist 1 (AUC = 0.639; 95% CI: 0.553, 0.719; *p* = 0.093), and Radiologist 2 (AUC = 0.677; 95% CI: 0.592, 0.754; *p* = 0.194).

**Table 4:** Diagnostic performances and pairwise comparisons (DeLong’s test) of Radiomics, O-RADS, and Radiologists’ Assessment for classifying benign and malignant ovarian masses in solid, cystic-solid and cystic subgroups

Model	Solid (*n* = 101)				Cystic-solid (*n* = 258)				Cystic (*n* = 138)			
	*p-*value	AUC	Sensitivity	Specificity	*p-*value	AUC	Sensitivity	Specificity	*p-*value	AUC	Sensitivity	Specificity
**Radiomics**		0.921 (0.850–0.965)	0.908 (0.819–0.962)	0.880 (0.688–0.975)		0.975 (0.948–0.990)	0.919 (0.867–0.957)	0.958 (0.897–0.989)		0.848 (0.777–0.903)	0.700 (0.348–0.933)	0.953 (0.901–0.983)
vs. O-RADS	0.002				0.332				0.542			
vs. Radiologist 1	0.001				*<* 0.001				0.093			
vs. Radiologist 2	0.028				0.008				0.194			
**O-RADS**		0.756 (0.661–0.836)	0.658 (0.540–0.763)	0.880 (0.688–0.975)		0.965 (0.935–0.984)	0.883 (0.823–0.928)	0.917 (0.842–0.963)		0.776 (0.633–0.789)	0.600 (0.262–0.878)	0.898 (0.833–0.964)
vs. Radiologist 1	0.442				*<* 0.001				0.321			
vs. Radiologist 2	0.236				0.003				0.184			
**Radiologist 1**		0.712 (0.613–0.798)	0.553 (0.434–0.667)	0.800 (0.593–0.932)		0.898 (0.855–0.932)	0.796 (0.726–0.855)	0.854 (0.767–0.918)		0.639 (0.553–0.719)	0.500 (0.187–0.813)	0.844 (0.769–0.902)
vs. Radiologist 2	0.009				0.009				0.755			
**Radiologist 2**		0.822 (0.733–0.891)	0.645 (0.527–0.751)	0.880 (0.688–0.975)		0.935 (0.898–0.962)	0.809 (0.740–0.866)	0.885 (0.804–0.941)		0.677 (0.592–0.754)	0.800 (0.444–0.975)	0.563 (0.472–0.650)

Data in parentheses are 95% confidence intervals

*AUC* area under the receiver operating characteristic curve, *O-RADS* Ovarian-Adnexal Reporting and Data System

The radiomics model demonstrated consistent performance across different scanner types and field strengths. When stratified by field strength, the AUCs were 0.959 (95% CI: 0.931, 0.978) for 3.0 T scanners (*n* = 327) and 0.958 (95% CI: 0.916, 0.983) for the 1.5 T scanner (*n* = 170), with no significant difference (*p* = 0.953). Similarly, pairwise comparisons between individual scanners showed no significant differences: GE 3.0 T vs. Philips 3.0 T (*p* = 0.204), GE 3.0 T vs. Philips 1.5 T (*p* = 0.139), and Philips 3.0 T vs. Philips 1.5 T (*p* = 0.538). Detailed results are provided in Supplementary Tables S6 and S7.

## Discussion

Accurate characterization of ovarian masses is crucial for optimal patient treatment. In this study, the radiomics model was developed, and its diagnostic performance was compared with that of the O-RADS and radiologists’ subjective assessment. The proposed radiomics model, which incorporates DWI, ADC, T2WI-FS, and CE-T1WI, demonstrates effective identification of malignant ovarian masses. The diagnostic performance of the radiomics model was higher than or comparable with that of radiologists and the O-RADS in differentiating malignant from benign ovarian masses. Specifically, the radiomics model allows for further refined risk stratification in ovarian masses of O-RADS score 4. In addition, in the subgroups of solid and cystic–solids masses, the radiomics model exhibited excellent performance, with significant improvements compared with that of the O-RADS and radiologists’ assessment. These findings highlight the potential of radiomics to serve as a decision-making adjunct in clinical practice. By providing quantitative and reproducible risk stratification, the radiomics model could reduce diagnostic uncertainty in ambiguous cases and guide personalized management strategies—such as selecting patients for conservative monitoring vs. surgical intervention.

Previous studies have underlined the value of radiomics models in classifying ovarian masses [19–28]. While existing models demonstrate promising results, our approach distinguishes itself through two key methodological advancements. First, in contrast to prior radiomics models that relied on limited sequences (e.g., dynamic contrast-enhanced MRI pharmacokinetic protocol [19]), our model integrates complementary information from four sequences (DWI, ADC, T2WI-FS, and CE-T1WI). This multi-parametric design captures both functional (DWI/ADC) and anatomical (T2WI-FS/CE-T1WI) tumor characteristics, mimicking the radiologists’ comprehensive diagnostic workflow. For instance, Zhang et al [20] achieved an AUC of 0.967 using axial T1WI, coronal T2WI, sagittal T2WI-FS, and ADC, but their model omitted contrast-enhanced sequences critical for vascularity assessment. Second, unlike many AI-based approaches trained on homogeneous single-scanner data [19, 20, 22], our model was rigorously validated across multi-vendor scanners (GE and Philips) and field strengths (1.5 T and 3.0 T). This heterogeneity, often overlooked in prior work [21, 25], ensures robustness to real-world imaging variability. For example, the model maintained comparable performance between 3.0 T (AUC 0.959) and 1.5 T (AUC 0.0.958; *p* = 0.953).

The O-RADS MRI has been validated as an effective tool for differentiating adnexal masses [15]. In our study, the O-RADS exhibited high diagnostic efficiency in the training set (152 benign masses and 141 malignant masses), with an AUC of 0.917. In the previous study, Wu et al evaluated the performance of the O-RADS in diagnosing adnexal masses, which included 362 adnexal masses (320 benign masses and 42 malignant masses), reporting an AUC of 0.918 [31], similar to our study.

Despite the excellent performance and reproducibility of the O-RADS, the positive predictive value for malignancy in O-RADS category 4 lesions is approximately 50% [14], which offers limited information. This may lead to unnecessary resection of benign ovarian masses, potentially affecting the fertility of premenopausal women, or result in suboptimal surgical treatment of malignant tumors. Therefore, it is necessary to further classify the risk of the O-RADS score 4. Recent research suggests that ADC analysis of solid tissue in adnexal masses can aid in differentiating invasive masses within O-RADS score 4 [32]. Notably, our study is the first research to extend risk stratification for the O-RADS score of 4 based on radiomics, and the radiomics achieved an AUC of 0.879 in categorizing benign and malignant ovarian masses, significantly outperforming radiologists, which could directly inform clinical decision-making. For instance, low-risk predictions may justify conservative management (e.g., short-interval MRI follow-up), preserving fertility in young patients, while high-risk predictions could prioritize surgical referral with gynecologic oncology consultation.

It is imperative to differentiate between benign and malignant ovarian masses with different morphological characteristics. Previous studies have indicated that MR spectroscopy and DWI can differentiate between benign and malignant solid ovarian masses [33, 34]. In the subgroups of solid and cystic–solids masses, our results demonstrated the highest AUCs of radiomics in differentiating between benign and malignant ovarian masses, with statistically significant improvements compared with those of radiologists, which suggests that radiomics can assist radiologists in classifying solid and cystic–solids ovarian masses as benign or malignant.

To facilitate clinical integration, future implementations could leverage semi-automated segmentation tools with deep learning-based contouring, reducing manual annotation time. However, translating radiomics into practice requires addressing regulatory (e.g., FDA certification) and computational hurdles (e.g., GPU/cloud infrastructure), alongside interoperability with PACS/RIS via DICOM standards.

Several limitations should be mentioned. First, the retrospective design and relatively small sample size from two centers may limit generalizability, particularly across diverse populations. However, validation across multi-vendor MRI scanners (GE 3.0 T, Philips 3.0 T/1.5 T) partially mitigates technical heterogeneity (Supplementary Table S6 and S7). In addition, the O-RADS assessments were based on non-DCE protocols, which may reduce their diagnostic accuracy as per the original O-RADS guidelines and hinder direct comparison with radiomics features derived from DCE imaging. Finally, in the subgroup analyses, the number of malignant masses was lower in the subgroup of cystic masses, and the number of benign and malignant masses was uneven. Future studies should prioritize prospective multi-center validation across diverse populations to confirm generalizability. Additionally, cost-effectiveness analyses are essential to evaluate the clinical feasibility of integrating radiomics into routine workflows, balancing diagnostic accuracy against resource utilization (e.g., segmentation time, computational infrastructure). Finally, incorporating DCE-MRI into both O-RADS and radiomics protocols may improve diagnostic concordance and adherence to guideline recommendations

In summary, the performance of the radiomics model to categorize ovarian masses was superior to O-RADS and junior radiologists and similar to senior radiologists. Furthermore, as a complementary tool to O-RADS, it allows for refined risk stratification for ovarian masses with an O-RADS score of 4 and different morphological characteristics, providing clinicians with quantitative decision support to improve preoperative diagnosis and guide treatment planning.

## Supplementary information

ELECTRONIC SUPPLEMENTARY MATERIAL

## Data Availability

The datasets used and/or analyzed during the current study are available from the corresponding author upon reasonable request.

## Abbreviations

ADC: Apparent diffusion coefficient
AUC: Area under the receiver operating characteristic curve
CA-125: Cancer antigen 125
CE-T1WI: Contrast-enhanced T1-weighted imaging
CI: Confidence interval
DWI: Diffusion-weighted magnetic resonance imaging
ICC: Intraclass correlation coefficient
O-RADS: Ovarian-Adnexal Reporting and Data System
ROC: Receiver operating characteristic curve
T2WI-FS: T2-weighted imaging with fat suppression
VOI: Volume of interest
