# Supplementary material for: The value of a radiomics model in predicting ovarian malignancy: a retrospective multi-center comparison with O-RADS and radiologists
Source: Insights Imaging. 2025 Jul 31;16:163. doi: 10.1186/s13244-025-02047-w (PMC12314133; doi:10.1186/s13244-025-02047-w)
Supplement: Supplementary file 1 — ELECTRONIC SUPPLEMENTARY MATERIAL [file 13244_2025_2047_MOESM1_ESM.pdf]

The Value of a Radiomics Model in Predicting Ovarian Malignancy: A Retrospective Multi-Center Comparison with O-RADS and Radiologists

ELECTRONIC SUPPLEMENTARY MATERIAL

Supplementary Note: Radscore formula

Radscore = 15.568138897641 + -0.0586555052915068 * T1E_wavelet.LHL_firstorder_Kurtosis + 0.0165419902685519 * T1E_exponential_glszm_GrayLevelNonUniformity + 4.78211327920152 * T1E_exponential_glcm_Imc2 + 9.07868592894721 * ADC_wavelet.HLL_glcm_Correlation + 11.2731952757816 * T1E_wavelet.LLH_glcm_Correlation + -9.45962329364552e-05 * DWI_exponential_glrlm_RunLengthNonUniformity + -0.152739153002883 * ADC_square_firstorder_Kurtosis + -22.8567481582121 * T1E_log.sigma.1.mm.3D_glcm_Correlation + -37.2318299322649 * T1E_square_glszm_LowGrayLevelZoneEmphasis + -16.2562818356195 * T1E_exponential_glcm_Idm + -523.432899659358 * DWI_wavelet.LLL_glszm_LowGrayLevelZoneEmphasis + -0.221359285377884 * T1E_exponential_firstorder_Median + 1.80876963033003 * T1E_square_ngtdm_Contrast + -514.66835103789 * T2_square_glcm_MaximumProbability + 0.00778042318534401 * T1E_exponential_glrlm_RunVariance + 18.1007454310545 * T1E_wavelet.HHH_glcm_Correlation

Table S1.MRI protocols of 3.0-T GE SIGNA Premier scanner.

Sequences Parameters	Axial T1WI	Axial T2WI	Axial T2WI FS	Sagittal T2WI	Coronal T2WI	DWI	Axial CE-T1WI
TR(msec)	624	5999	4829	4719	4586	4500	3.3
TE(msec)	8	102	85	85	102	60	1.5
Slice thickness(mm)	6	6	6	5	5	3	1.5
Acquisition matrix	384×384	384×384	384×384	400×320	384×384	84×64	320×320
FOV(mm)	380×380	380×380	380×380	240×240	260×260	160×80	380×380
Interslice gap	1	1	1	1	0.5	0.3	0
NSA	1	1	1	1	1	1	1

NOTE: FS, fat suppression; FOV, field of view; NSA, number of signals averaged; CE, contrast-enhanced.

Table S2.MRI protocols of 3.0-T Philips Ingenia DNA scanner.

Sequences Parameters	Axial T1WI	Axial T2WI	Axial T2WI FS	Sagittal T2WI	Coronal T2WI	DWI	Axial CE-T1WI
TR(msec)	464	3078	3167	3699	3000	2628	3.7
TE(msec)	8	80	75	90	90	80	1.31
Slice thickness(mm)	8	8	8	4	5	8	3.5
Acquisition matrix	296×375	312×366	280×361	252×252	376×370	124×106	252×180
FOV(mm)	250×380	250×379	250×380	250×250	300×380	375×315	400×318
Interslice gap	0.8	0.8	0.8	0.4	0.5	0.8	-1.75
NSA	1	1	1	1	1	1	1

NOTE: FS, fat suppression; FOV, field of view; NSA, number of signals averaged; CE, contrast-enhanced.

Table S3.MRI protocols of 1.5-T Philips Ingenia XD scanner.

Sequences Parameters	Axial T1WI	Axial T2WI	Axial T2WI FS	Sagittal T2WI	Coronal T2WI	DWI	Axial CE-T1WI
TR(msec)	520	3000	3367	4722	4303	2397	5.5
TE(msec)	10	100	80	100	90	71	1.73
Slice thickness(mm)	8	8	8	4	5	8	3.5
Acquisition matrix	240×330	280×411	280×317	252×252	388×319	64×105	252×180
FOV(mm)	240×382	250×378	250×378	250×250	350×378	251×411	400×318
Interslice gap	0.8	0.8	0.8	0	0.5	0.8	-1.75
NSA	1	1	1	1	1	1	1

NOTE: FS, fat suppression; FOV, field of view; NSA, number of signals averaged; CE, contrast-enhanced.

Table S4. Histology of the ovarian masses

Pathology	No（%）
**Benign masses (n =249)**	
Mature teratoma	88 (17.7)
Serous cystadenoma	50 (10.1)
Endometriotic cyst	38 (7.7)
Mucinous cystadenoma	25 (5.0)
Thecoma	11 (2.2)
Seromucinous cystadenoma	10 (2.0)
Fibroma	8 (1.6)
Cellular fibroma	8 (1.6)
Serous adenofibroma	6 (1.2)
Brenner tumor	5 (1.0)
**Malignant masses (n=218)**	
High-grade serous carcinoma	141（28.4）
Clear cell carcinoma	25 (5.0)
Endometroid adenocarcinoma	13 (2.6)
Low-grade serous ovarian cancer	13 (2.6)
Immature teratoma	12 (2.4)
Mucinous adenocarcinoma	9 (1.8)
Adenocarcinoma	5 (1.0)
**Borderline masses(n=30)**	
Borderline serous tumor	13 (2.6)
Borderline mucinous tumor	8 (1.6)
Borderline seromucinous cystadenoma	6 (1.2)
Borderline endometrioid tumor	3 (0.6)

Table S5. The calibration metrics of radiomics model in training, internal and external validation cohorts.

	Training set	Internal validation set	External validation set
**Slope**	0.98 (0.95-0.1.02)	0.92 (0.88-0.97)	0.89 (0.84-0.94)
**Intercept**	-0.05	-0.08	-0.12
**Hosmer-Lemeshow test**	*P*=0.32	*P*=0.21	*P*=0.15
**Brier Score**	0. 086	0.102	0.118

Note.-Data in parentheses are 95% confidence intervals.

Table S6. Diagnostic performance and pairwise comparisons (DeLong's test) of Radiomics for differentiating benign and malignant ovarian masses at 3.0-T and 1.5-T MRI.

	3.0-T MRI	1.5-T MRI	*P*-value
**Number of Masses**			
-Benign	166	83	
-Malignant	161	87	
**AUC**	0.959 (0.931-0.978)	0.958 (0.916-0.983)	0.953
**Sensitivity**	0.926 (0.873-0.961)	0.851 (0.758-0.918)	
**Specificity**	0.934 (0.885-0.966)	0.964 (0.898-0.992)	

Note.-Data in parentheses are 95% confidence intervals. AUC=area under the receiver operating characteristic curve.

Table S7. Diagnostic performance and pairwise comparisons (DeLong's test) of radiomics for differentiating benign and malignant ovarian masses across multi-vendor MRI Scanners.

	**GE SIGNA Premier 3.0T**	**Philips Ingenia DNA 3.0T**	**Philips Ingenia XD 1.5T**	***P*-value**
**Number of Masses**				
-Benign	118	48	83	
-Malignant	129	32	87	
**AUC**	0.979 (0.953-0.993)	0.939 (0.863-0.981)	0.958 (0.916-0.983)	
GE 3.0T vs Philips 3.0T				0.204
GE 3.0T vs Philips 1.5T				0.139
Philips 3.0T vs Philips 1.5T				0.538
**Sensitivity**	0.977 (0.934-0.995)	0.875 (0.710-0.965)	0.851 (0.758-0.918)	
**Specificity**	0.915 (0.850-0.959)	0.917 (0.800-0.977)	0.964 (0.898-0.992)	

Table S8. Diagnostic performances and pairwise comparisons (DeLong's test) of Radiomics, O-RADS, and Radiologists' assessment for classifying benign and malignant ovarian masses in the O-RADS score 4 subgroup: internal and external validation cohorts.

Model	*P* value	AUC	Sensitivity	Specificity
**Radiomics**		0.873 (0.732-0.956)	0.852 (0.663-0.958)	0.857 (0.572-0.982)
vs Radiologist 1	0.014			
vs Radiologist 2	0.557			
**Radiologist 1**		0.627 (0.462-0.773)	0.222 (0.086-0.423)	1.000 (0.768-1.000)
vs Radiologist 2	0.005			
**Radiologist 2**		0. 829 (0.680-0.929)	0.593 (0.388-0.776)	1.000 (0.768-1.000)

Note.-Data in parentheses are 95% confidence intervals. AUC=area under the receiver operating characteristic curve, O-RADS=Ovarian-Adnexal Reporting and Data System. Subgroup sample sizes: n=41, 14 benign masses and 27 malignant masses.

Table S9. Diagnostic performances and pairwise comparisons (DeLong's test) of Radiomics, O-RADS, and Radiologists' Assessment for classifying benign and malignant ovarian masses in solid, cystic-solid and cystic subgroups: internal and external validation cohorts.

Model	Solid (n=41)				Cystic-solid (n=95)				Cystic (n=68)			
	P value	AUC	Sensitivity	Specificity	*P* value	AUC	Sensitivity	Specificity	*P* value	AUC	Sensitivity	Specificity
**Radiomics**		0.897 (0.761-0.970)	0.839 (0.663-0.945)	0.900 (0.555-0.997)		0.969 (0.911-0.994)	0.917 (0.816-0.972)	0.914 (0.769-0.982)		0.841 (0.732-0.919)	0.800 (0.284-0.995)	0.968 (0.890-0.996)
vs O-RADS	0.053				0.613				0.391			
vs Radiologist 1	0.019				0.004				0.442			
vs Radiologist 2	0.329				0.103				0.321			
**O-RADS**		0.766 (0.608-0.884)	0.613 (0.422-0.782)	1.000 (0.692-1.000)		0.961 (0.900-0.990)	0.850 (0.734-0.929)	0.943 (0.808-0.993)		0.717 (0.595-0.820)	0.400 (0.053-0.853)	0.921 (0.824-0.974)
vs Radiologist 1	0.173				0.003				0.794			
vs Radiologist 2	0.470				0.096				0.464			
**Radiologist 1**		0.650 (0.485-0.792)	0.258 (0.119-0.446)	1.000 (0.692-1.000)		0.881 (0.798-0.938)	0.750 (0.621-0.853)	0.886 (0.733-0.968)		0.675 (0.550-0.783)	0.600 (0.147-0.947)	0.730 (0.603-0.834)
vs Radiologist 2	0.004				0.052				0.791			
**Radiologist 2**		0.815 (0.662-0.918)	0.516 (0.331-0.698)	1.000 (0.692-1.000)		0.929 (0.858-0.972)	0.783 (0.658-0.879)	0.943 (0.808-0.993)		0.619 (0.493-0.734)	0.800 (0.284-0.995)	0.524 (0.394-0.651)

Note.-Data in parentheses are 95% confidence intervals. AUC=area under the receiver operating characteristic curve, O-RADS=Ovarian-Adnexal Reporting and Data System. Subgroup sample sizes: solid mass（10 benign, 31 malignant）, cystic-solid mass（35 benign, 60 malignant）, cystic mass（63 benign, 5 malignant）.

Table S10. Diagnostic performances and pairwise comparisons (DeLong's test) of Radiomics, O-RADS, and Radiologists' assessment for classifying benign and malignant ovarian masses in the O-RADS score 4 subgroup: training, internal and external validation cohorts.

Model	Training set (n=71)				Internal validation set (n=25)				External validation set（n=16）			
	P value	AUC	Sensitivity	Specificity	*P* value	AUC	Sensitivity	Specificity	*P* value	AUC	Sensitivity	Specificity
**Radiomics**		0.881 (0.783-0.946)	0.889 (0.774-0.958)	0.824 (0.566-0.962)		0.912 (0.729-0.988)	0.947 (0.740-0.999)	0.833 (0.259-0.996)		0.828 (0.561-0.967)	0.750 (0.349-0.968)	0.875 (0.473-0.997)
vs Radiologist 1	0.002				0.150				0.029			
vs Radiologist 2	0.034				0.688				0.707			
**Radiologist 1**		0.611 (0.488-0.725)	0.241 (0.135-0.376)	1.000 (0.805-1.000)		0.711 (0.496-0.873)	0.526 (0.289-0.756)	0.833 (0.359-0.996)		0.547 (0.285-0.791)	0.250 (0.142-0.651)	1.000 (0.631-1.000)
vs Radiologist 2	0.127				0.064				0.073			
**Radiologist 2**		0.708 (0.588-0.810)	0.574 (0.432-0.708)	0.824 (0.566-0.962)		0.873 (0.679-0.971)	0.579 (0.335-0.797)	1.000 (0.541-1.000)		0.781 (0.509-0.944)	0.625 (0.245-0.915)	1.000 (0.631-1000)

Note.-Data in parentheses are 95% confidence intervals. AUC=area under the receiver operating characteristic curve, O-RADS=Ovarian-Adnexal Reporting and Data System. Subgroup sample sizes: training set（17 benign, 54 malignant）, internal validation set（6 benign, 19 malignant）, external validation set（8 benign, 8 malignant）.

Table S11. Diagnostic performances and pairwise comparisons (DeLong's test) of Radiomics, O-RADS, and Radiologists' Assessment for classifying benign and malignant ovarian masses in solid, cystic-solid and cystic subgroups: training cohort.

Model	Solid (n=60)				Cystic-solid (n=163)				Cystic (n=70)			
	P value	AUC	Sensitivity	Specificity	*P* value	AUC	Sensitivity	Specificity	*P* value	AUC	Sensitivity	Specificity
**Radiomics**		0.947 (0.856-0.988)	0.956 (0.849-0.995)	0.867 (0.595-0.983)		0.980 (0.945-0.996)	0.941 (0.876-0.978)	0.967 (0.887-0.996)		0.874 (0.773-0.941)	1.000 (0.478-1.000)	0.662 (0.534-0.774)
vs O-RADS	0.010				0.297				0.756			
vs Radiologist 1	0.004				0.002				0.310			
vs Radiologist 2	0.026				0.031				0.287			
**O-RADS**		0.747 (0.618-0.850)	0.689 (0.534-0.818)	0.800 (0.519-0.957)		0.966 (0.925-0.988)	1.000(0.964-1.000)	0.820 (0.700-0.906)		0.815 (0.705-0.898)	0.800 (0.284-0.995)	0.923 (0.830-0.975)
vs Radiologist 1	0.929				0.003				0.504			
vs Radiologist 2	0.269				0.018				0.279			
**Radiologist 1**		0.753 (0.625-0.856)	0.533 (0.379-0.683)	0.933 (0.681-0.998)		0.910 (0.856-0.949)	0.824 (0.736-0.892)	0.836(0.719-0.918)		0.926 (0.838-0.975)	1.000 (0.478-1.000)	0.785 (0.665-0.877)
vs Radiologist 2	0.128				0.108				0.083			
**Radiologist 2**		0.836 (0.717-0.919)	0.556 (0.400-0.704)	1.000 (0.782-1.000)		0.939 (0.890-0.970)	1.000 (0.964-1.000)	0.705 (0.574-0.815)		0.734 (0.615-0.832)	0.800 (0.284-0.995)	0.600 (0.471-0.720)

Note.-Data in parentheses are 95% confidence intervals. AUC=area under the receiver operating characteristic curve, O-RADS=Ovarian-Adnexal Reporting and Data System. Subgroup sample sizes: solid mass（15 benign, 45 malignant）, cystic-solid mass（61 benign, 102 malignant）, cystic mass（65 benign, 5 malignant）.

Table S12. Diagnostic performances and pairwise comparisons (DeLong's test) of Radiomics, O-RADS, and Radiologists' Assessment for classifying benign and malignant ovarian masses in solid, cystic-solid and cystic subgroups: internal validation cohort.

Model	Solid (n=28)				Cystic-solid (n=61)				Cystic (n=35)			
	***P* value**	**AUC**	**Sensitivity**	**Specificity**	***P* value**	**AUC**	**Sensitivity**	**Specificity**	***P* value**	**AUC**	**Sensitivity**	**Specificity**
**Radiomics**		0.924 (0.759-0.990)	0.909 (0.708-0.989)	0.833 (0.359-0.996)		0.986 (0.915-1.000)	0.925 (0.796-0.984)	1.000 (0.839-1.000)		0.970 (0.848-0.999)	1.000 (0.158-1.000)	0.939 (0.798-0.993)
vs O-RADS	0.190				0.126				1.000			
vs Radiologist 1	0.162				0.008				0.080			
vs Radiologist 2	0.400				0.065				<0.001			
**O-RADS**		0.807 (0.614-0.930)	0.682 (0.451-0.861)	1.000 (0.541-1.000)		0.949 (0.861-0.989)	0.825 (0.672-0.927)	0.952 (0.762-0.999)		0.970 (0.848-0.999)	1.000 (0.158-1.000)	0.939 (0.798-0.993)
vs Radiologist 1	0.532				0.040				0.128			
vs Radiologist 2	0.567				0.421				<0.001			
**Radiologist 1**		0.754 (0.555-0.896)	0.636 (0.407-0.828)	0.833 (0.359-0.996)		0.871 (0.760-0.943)	0.750 (0.588-0.873)	0.857(0.637-0.970)		0.636 (0.457-0.792)	0.500 (0.478-1.000)	0.758 (0.577-0.889)
vs Radiologist 2	0.102				0.092				0.686			
**Radiologist 2**		0.848 (0.663-0.955)	0.773 (0.546-0.922)	0.833 (0.359-0.996)		0.929 (0.834-0.979)	0.775 (0.615-0.892)	0.952 (0.762-0.999)		0.727 (0.551-0.863)	1.000 (0.158-1.000)	0.456 (0.281-0.636)

Note.-Data in parentheses are 95% confidence intervals. AUC=area under the receiver operating characteristic curve, O-RADS=Ovarian-Adnexal Reporting and Data System. Subgroup sample sizes: solid mass（6 benign, 22 malignant）, cystic-solid mass（21 benign, 40 malignant）, cystic mass（33 benign, 2 malignant）.

Table S13. Diagnostic performances and pairwise comparisons (DeLong's test) of Radiomics, O-RADS, and Radiologists' Assessment for classifying benign and malignant ovarian masses in solid, cystic-solid and cystic subgroups: external validation cohort.

Model	Solid (n=13)				Cystic-solid (n=34)				Cystic (n=33)			
	P value	AUC	Sensitivity	Specificity	*P* value	AUC	Sensitivity	Specificity	*P* value	AUC	Sensitivity	Specificity
**Radiomics**		0.833 (0.531-0.976)	0.667 (0.299-0.925)	1.000 (0.398-1.000)		0.964 (0.837-0.998)	1.000 (0.832-1.000)	0.929 (0.661-0.998)		0.844 (0.676-0.947)	0.667 (0.094-0.992)	1.000 (0.884-1.000)
vs O-RADS	0.190				0.569				0.054			
vs Radiologist 1	0.250				0.110				0.600			
vs Radiologist 2	0.628				0.393				0.393			
**O-RADS**		0.667 (0.362-0.894)	0.444 (0.137-0.788)	1.000 (0.398-1.000)		0.979 (0.859-1.000)	0.900 (0.683-0.988)	0.929 (0.661-0.998)		0.517 (0.337-0.693)	1.000 (0.292-1.000)	0.200 (0.077-0.386)
vs Radiologist 1	0.597				0.030				0.256			
vs Radiologist 2	0.614				0.144				0.852			
**Radiologist 1**		0.556 (0.265-0.820)	0.556 (0.212-0.863)	0.750 (0.194-0.994)		0.902 (0.750-0.977)	0.750 (0.509-0.913)	0.929 (0.661-0.998)		0.700 (0.516-0.846)	0.667 (0.094-0.992)	0.700 (0.506-0.853)
vs Radiologist 2	0.467				0.377				0.677			
**Radiologist 2**		0.750 (0.442-0.940)	0.444 (0.137-0.788)	1.000 (0.398-1.000)		0.932 (0.791-0.943)	0.800 (0.563-0.892)	0.929 (0.661-0.998)		0.561 (0.378-0.733)	0.667 (0.094-0.992)	0.600 (0.406-0.773)

Note.-Data in parentheses are 95% confidence intervals. AUC=area under the receiver operating characteristic curve, O-RADS=Ovarian-Adnexal Reporting and Data System. Subgroup sample sizes: solid mass（4 benign, 9 malignant）, cystic-solid mass（14 benign, 20 malignant）, cystic mass（30 benign, 3 malignant）.
